# Supplementary material for: Mechanical properties of mandibular and maxillary bone collagen fibrils based on nonlocal elasticity theory
Source: Biophys Rep (N Y). 2025 Apr 17;5(2):100210. doi: 10.1016/j.bpr.2025.100210 (PMC12138556; doi:10.1016/j.bpr.2025.100210)
Supplement: Document S1. Supporting material [file mmc1.pdf]

Biophysical Reports, Volume *5*

Supplemental information

Mechanical properties of mandibular and maxillary bone collagen fibrils based on nonlocal elasticity theory

Elaheh Alibeigi Beni, Alireza Shahidi, and Behnaz Ebadian

Determination of indentation modulus

According to the Oliver-Pharr method, contact stiffness can be calculated from the unloading part (Fig. 4b) of the force-indentation curve. In this regard, the following equation is fitted on the unloading force–indentation data[22].

$$P = a\left(h - h_f\right)^m \quad (1)$$

, where P is the indenter force. Also, h, h_f stand for the indentation and final indentation depth, respectively; a and m are the fitting parameters as well. h_c represents the contact depth as determined by the following equation.

$$h_c = h_{\max} - \varepsilon \frac{P_{\max}}{S_c} \quad (2)$$

, where the contact stiffness, S_c, is the slope of the unloading curve at the maximum indentation depth. In this equation, ε is a constant parameter and a function of the exponent m that depends on the geometry of the tip[1].

$$\varepsilon(m) = m\left[1 - \frac{2\Gamma(m/2(m-1))}{\sqrt{\pi}\,\Gamma(1/2(m-1))}(m-1)\right] \quad (3)$$

In the forgoing equation, Γ stands for Gamma function. The reduced modulus from indentation experiments is calculated according to the following equation [22].

$$E_r = \frac{\sqrt{\pi} S_c}{2\sqrt{A_c}} \quad (4)$$

Fig. 5. shows the indentation parameters[31].
The contact area is determined as follows[31]:

$$A_c = \pi \tan^2(\theta) h_c^2 \quad (5)$$

The reduced modulus is a combination of E_{sample}, the elastic modulus of the sample and $E_{indenter}$ the elastic modulus of the indenter.,

$$\frac{1}{E_r} = \frac{1 - \nu_{sample}^2}{E_{sample}} + \frac{1 - \nu_{indenter}^2}{E_{indenter}} \quad (6)$$

In this equation, ν_{sample}, $\nu_{indenter}$ stand for the Poisson's ratio of the sample and indenter, respectively. In the case of, the tip is much stiffer than the sample, and the second term of Eq. (6) is close to zero. Therefore, the equation is rewritten as follows:

$$\frac{1}{E_r} = \frac{1 - \nu_{sample}^2}{E_{sample}} \quad (7)$$

The distribution of stress and strain

The distribution of stress and strain in a semi-infinite elastic medium, when it is deformed by a rigid conical indenter, is determined. According to Fig. 5. the position of a point in the medium is described by cylindrical polar coordinates (r, θ, z). Due to the symmetry around the z-axis, the

displacement vector is considered as the form $(u_r, 0, u_z)$. Here, u_r and u_z represent the displacement along the r-axis and z-axis, respectively[23].
In addition, $\sigma_r, \sigma_\theta, \sigma_z, \tau_{rz}$ stand for the components of the stress tensor. It should be noted that other components of the stress tensor, $\tau_{r\theta}, \tau_{z\theta}$, are equal to zero at any point of the solid.
Considering the equations of elastic equilibrium, it is as follows:

$$\begin{aligned} &\frac{\partial \sigma_r}{\partial r} + \frac{\partial \tau_{rz}}{\partial z} + \frac{1}{r}(\sigma_r - \sigma_\theta) = 0 \\ &\frac{\partial \tau_{rz}}{\partial r} + \frac{\partial \sigma_z}{\partial z} + \frac{\tau_{rz}}{r} = 0 \end{aligned} \tag{8}$$

Over the entire surface $z = 0$, the shear stress equals zero. The boundary conditions are assumed to be as follows:

$$\begin{aligned} &[\sigma_z(r)]_{z=0} = 0 \qquad (z = 0, \quad r > r_c) \\ &[\tau_{rz}(r)]_{z=0} = 0 \qquad (z = 0, \quad r \geq 0) \end{aligned} \tag{9}$$

Eqs. (10-15) can satisfy the equations of elastic equilibrium and boundary conditions[23].

$$\frac{r_c \sigma_z}{h_c E} = -\frac{1}{2(1-\nu^2)}\left[J_1^0(\rho,\xi) + \xi\, J_2^0(\rho,\xi)\right] \tag{10}$$

$$\frac{r_c \tau_{zr}}{h_c E} = -\frac{1}{2(1-\nu^2)}\xi\, J_2^1(\rho,\xi) \tag{11}$$

$$\frac{r_c \sigma_\theta}{h_c E} = -\frac{1}{2(1-\nu^2)}\left[(2\nu)J_1^0(\rho,\xi) + \frac{1}{\rho}\left[(1-2\nu)J_0^1(\rho,\xi) - \xi J_1^1(\rho,\xi)\right]\right] \tag{12}$$

$$\frac{r_c(\sigma_r + \sigma_\theta + \sigma_z)}{h_c E} = -\frac{1}{(1-\nu)} J_1^0(\rho,\xi) \tag{13}$$

$$u_r = h_c\left(\frac{1-2\nu}{2(1-\nu)}J_0^1 - \frac{\xi}{2(1-\nu)}J_1^1\right) \tag{14}$$

$$u_z = -h_c\left(\frac{\xi}{2(1-\nu)}J_1^0 + J_0^0(\rho,\xi)\right) \tag{15}$$

In Eqs. (10-13), E and ν represent elastic modulus and Poisson's ratio, respectively. $J_n^m(\rho,\xi)$ is also defined as follows:

$$J_n^m(\rho,\xi) = \int_0^\infty (1-\cos(p))p^{n-2} J_m(\rho p) e^{-p\xi} dp \qquad \rho = \frac{r}{r_c}, \quad \xi = \frac{z}{r_c}, \tag{16}$$

$J_m(\rho p)$ stands for Bessel's integral.
As the first step, to determine the distribution of stress and strain along the axis of symmetry, ρ is set to zero.

$$J_1^0(0,\xi) = \int_0^\infty \frac{1-\cos p}{p} e^{-p\xi} dp = \int_0^\infty \int_0^1 \sin(tp) e^{-p\xi} dt dp = \frac{1}{2}\ln\left(1+\frac{1}{\xi^2}\right)$$

$$J_2^0(0,\xi) = \int_0^\infty (1-\cos p) e^{-p\xi} dp = \frac{1}{\xi(1+\xi^2)} \tag{17}$$

$$J_0^0(0,\xi) = \int_0^\infty \left(\frac{1-\cos p}{p^2}\right) e^{-\xi p} dp = \frac{-\pi}{4\xi} - \frac{\xi}{2}\ln\left(1+\frac{1}{\xi^2}\right) - \xi\left(\ln(p) - \sum_{n=0}^{\infty} \frac{(-\xi p)^n}{n(n!)}\right)_{p=0,\infty}$$

Also, the following result is obtained[23]:

$$\frac{2}{\rho} J_0^1(0,\xi) = J_1^0(0,\xi)$$

$$\frac{2}{\rho} J_1^1(0,\xi) = J_2^0(0,\xi) \tag{18}$$

By substituting Eqs. (17, 18) into Eqs. (10-13), the components of stress on the axis of symmetry are expressed as follows:

$$\frac{r_c \sigma_r}{h_c E} = -\frac{1}{4(1-\nu^2)}\left\{\left(\nu+\frac{1}{2}\right)\ln\left(1+\frac{1}{\xi^2}\right) - \frac{1}{1+\xi^2}\right\}$$

$$\frac{r_c \sigma_z}{h_c E} = -\frac{1}{2(1-\nu^2)}\left\{\frac{1}{2}\ln\left(1+\frac{1}{\xi^2}\right) + \frac{1}{1+\xi^2}\right\} \tag{19}$$

$$\sigma_r = \sigma_\theta$$

Given that $J_2^1(0,\xi)$ is equal to zero τ_{rz} is zero and $\sigma_r, \sigma_z, \sigma_\theta$ in Eq. (19) are the principal stresses at any point of the axis of symmetry. The principal shearing stress, τ , is calculated according to $1/2|\sigma_r - \sigma_z|$. Components of the strain tensor are determined by employing Eqs. (14, 15) and substituting Eq. (17), as follows:

$$\varepsilon_z = \frac{\partial u_z(0,\xi)}{\partial z} = -h_c\left(\left(\frac{1}{4r_c(1-\nu)} - \frac{1}{2r_c}\right)\left(\ln\left(1+\frac{1}{\xi^2}\right)\right) - \frac{1}{2r_c(1-\nu)}\left(\frac{1}{(1+\xi^2)}\right)\right)$$

$$\varepsilon_r = \frac{\partial u_r(0,\xi)}{\partial r} = h_c\left(\frac{1-2\nu}{8r_c(1-\nu)}\ln\left(1+\frac{1}{\xi^2}\right) - \frac{1}{4r_c(1-\nu)}\left(\frac{1}{(1+\xi^2)}\right)\right) \tag{20}$$

ε_{rz} is zero and $\varepsilon_r, \varepsilon_z$ in Eq. (20) are the principal strains at any point of the axis of symmetry. It must be noted that the principal shearing stress along the axis r=0, decreases steadily with increasing (ξ) and it is infinite at the origin of coordinates $\rho = \xi = 0$. Therefore, it can be concluded that the distribution of stress and strain at all points of the medium, except the immediate vicinity of the apex of the cone, are obtained by elastic solution. To determine the components of stress and strain tensor at any points in the interior of the elastic medium, the integrals $J_n^m(\rho,\xi)$ are expressed as the following equation[23].

$$J_n^m(\rho,\xi) = Z_n^m(\rho,\xi) - C_n^m(\rho,\xi) \qquad m+n \geq 2 \tag{21}$$

The first integral is evaluated as[23]:

$$Z_n^m(\rho,\xi)=\int_0^\infty p^{n-2}J_m(\rho p)e^{-p\xi}dp=\sum_{k=0}^{\infty}\frac{(-1)^k\left(\frac{1}{2}\rho\right)^{m+2k}}{k!\Gamma(m+k+1)}\int_0^\infty p^{n+m+2k-2}e^{-p\xi}dp$$
$$=\sum_{k=0}^{\infty}\frac{(-1)^k\left(\frac{1}{2}\rho\right)^{m+2k}}{k!\Gamma(m+k+1)}\frac{\Gamma(n+m+2k-1)}{\xi^{n+m+2k-1}} \quad (22)$$

Also, the second integral is defined as:

$$C_n^m(\rho,\xi)=\int_0^\infty p^{n-2}\cos p\, J_m(\rho p)e^{-p\xi}dp \quad (23)$$

By calculating the integrals $J_m^n(\rho,\xi)$ and substituting them into Eqs. (10-13), the components of the stress tensor in the interior of the elastic medium are obtained. The components of the strain tensor are calculated according to the Eq. (24). The details of the integrals $J_m^n(\rho,\xi)$ solution for different values of m and n are presented in Appendix A.

$$\varepsilon_z=\frac{\partial u_z(\rho,\,\xi)}{\partial z}=-h_c\left(\frac{-J_1^0(\rho,\,\xi)}{r_c}+\frac{J_1^0(\rho,\,\xi)}{2r_c(1-\nu)}-\frac{\xi\,J_2^0(\rho,\,\xi)}{2r_c(1-\nu)}\right)$$
$$\varepsilon_r=\frac{\partial u_r(\rho,\,\xi)}{\partial r}=h_c\left(\frac{1-2\nu}{2r_c(1-\nu)}\left(J_1^0(\rho,\xi)-\frac{J_0^1(\rho,\xi)}{\rho}\right)-\left(\frac{\xi}{2r_c(1-\nu)}\right)\left(J_2^0(\rho,\xi)-\frac{J_1^1(\rho,\xi)}{\rho}\right)\right)$$
$$\varepsilon_{rz}=\frac{\partial u_r(\rho,\,\xi)}{\partial z}+\frac{\partial u_z(\rho,\,\xi)}{\partial r}=h_c\left(\left(\frac{2\nu-1+\xi r_c}{2r_c(1-\nu)}\right)J_1^1(\rho,\xi)+\frac{\xi}{2r_c(1-\nu)}J_2^1(\rho,\xi)+J_0^1(\rho,\xi)\right) \quad (24)$$
$$\varepsilon_\theta=\frac{u_r(\rho,\,\xi)}{r}=\frac{h_c}{r}\left(\frac{1-2\nu}{2(1-\nu)}J_0^1(\rho,\xi)-\frac{\xi}{2(1-\nu)}J_1^1(\rho,\xi)\right)$$

Nonlocal elasticity theory at small scales

According to this theory, in the domain, the stress at the reference point is a function of the strains at all other points. The components of the stress tensor for a linear homogenous nonlocal elastic body, neglecting the body force, are expressed according to the following equation.

$$\sigma_{ij}(x)=\int\lambda(|x-x'|,\alpha)C_{ijkl}\varepsilon_{kl}(x')dV(x'),\quad \forall x\in V \quad (25)$$

In the forgoing equation, $\sigma_{ij},\varepsilon_{kl},C_{ijkl}$ represent stress, strain and fourth-order elasticity tensor, respectively. $\lambda(|x'-x|,\alpha)$ is also the nonlocal modulus and has a dimension of $(length)^{-3}$. x stands for a reference point at time t in the body. $|x'-x|$ is the distance in the Euclidean form. $(\alpha=e_0l_i/l_e)$ represents the scale coefficient or nonlocal parameter of the length unit. In fact, this parameter incorporates the effect of the nano-scale on mechanical behavior. l_i and l_e also

stand for internal characteristic lengths and external characteristic length, respectively. e_0 is a constant parameter that is appropriate for each material. In other words, this parameter should be determined for each nanostructure independently[18, 28]. Due to the difficulty of solving the integral constitutive relation, a differential form equation is used as shown in Eq. (26)[28]:

$$\left(1-\alpha^2 l_e^2 \nabla^2\right)\sigma = C:\varepsilon, \quad \alpha = e_0 l_i / l_e \tag{26}$$

In the forgoing equation, ':' represents the double dot product and $\nabla^2 = \frac{\partial^2}{\partial r^2} + \frac{1}{r}\frac{\partial}{\partial r} + \frac{\partial^2}{\partial z^2}$ refers to the laplacian operator. Collagen fibril is assumed to be a homogenous and isotropic solid. Poisson's ratio is also considered to be 0.5[29]. By considering Eqs. (10-13) and Eq. (24), and substituting the components of stress and strain tensor in Eq. (26) and applying the laplacian operator, nonlocal constitutive relations can be obtained. Finally, the nonlocal parameter $\left(\mu = e_0 l_i\right)$ can be obtained in the elastic medium.

Appendix A

In general, $C_m^n(\rho,\xi)$ is calculated by substituting $(\xi + i)$ for (ξ) in Eq. (23); afterward, the real part is equated.

$$Z_2^0(\rho,\xi) = \int_0^\infty e^{-p\xi} J_0(\rho p)\,dp = \frac{1}{\sqrt{\rho^2+\xi^2}} \tag{1-A}$$

In the following

$$C_2^0(\rho,\xi) = \int_0^\infty \cos p\, e^{-\xi p} J_0(\rho p)\,dp = \frac{\sqrt{2}}{2R}\sqrt{1+\frac{\rho^2+\xi^2-1}{R^2}} \tag{2-A}$$

$$R^4 = \left(\rho^2+\xi^2-1\right)^2 + 4\xi^2$$

$$J_2^0(\rho,\xi) = \frac{1}{\sqrt{\rho^2+\xi^2}} - \frac{\sqrt{2}}{2R}\sqrt{1+\frac{\rho^2+\xi^2-1}{R^2}} \tag{3-A}$$

To calculate $J_2^1(\rho,\xi)$, first, the following integral is calculated.

$$\begin{aligned}
&\int_0^\infty e^{-\xi p} J_n(\rho p)\,dp = \frac{1}{\pi}\int_0^\pi\int_0^\infty e^{-\xi p}\cos(n\theta - \rho p\sin\theta)\,dp\,d\theta = \\
&\frac{1}{\pi}\int_0^\pi\int_0^\infty e^{-\xi p}\cos(n\theta)\cos(\rho p\sin\theta)\,dp\,d\theta + \frac{1}{\pi}\int_0^\pi\int_0^\infty e^{-\xi p}\sin(n\theta)\sin(\rho p\sin\theta)\,dp\,d\theta = \\
&\frac{1}{\pi}\int_0^\pi \frac{-\xi\cos(n\theta)}{\xi^2+\rho^2\sin^2\theta}\,d\theta + \frac{1}{\pi}\int_0^\pi \frac{\rho\sin\theta\sin(n\theta)}{\xi^2+\rho^2\sin^2\theta}\,d\theta
\end{aligned} \tag{4-A}$$

Considering the $(n=1)$

$$Z_2^1(\rho,\xi) = \int_0^\infty e^{-\xi p} J_1(\rho p)\,dp = \frac{1}{\rho}\left(\frac{\xi}{\sqrt{\xi^2+\rho^2}} - 1\right) \tag{5-A}$$

Therefore, by substituting $(\xi + i)$ in Eq. (5-A) and calculating the real part

$$J_2^1(\rho,\xi)=\frac{\sqrt{2}}{2\rho R}\left(\xi\sqrt{1+\frac{\rho^2+\xi^2-1}{R^2}}+\sqrt{1-\frac{\rho^2+\xi^2-1}{R^2}}\right)-\frac{\xi}{\rho\sqrt{\rho^2+\xi^2}} \tag{6-A}$$

$Z_1^1(\rho,\xi)$ is obtained by applying Eq. (5-A) and integrating ξ

$$Z_1^1(\rho,\xi)=\int_0^\infty \frac{e^{-\xi p}}{p}J_1(\rho p)dp=\frac{1}{\rho}\left(\sqrt{\xi^2+\rho^2}-\xi\right) \tag{7-A}$$

In the forgoing equation, by substituting $(\xi+i)$ for (ξ), $C_1^1(\rho,\xi)$ is calculated by considering the real part of the expression

$$J_1^1(\rho,\xi)=\xi+\sqrt{\xi^2+\rho^2}-\frac{\sqrt{2}}{2}R\sqrt{1+\frac{\rho^2+\xi^2-1}{R^2}} \tag{8-A}$$

By integrating both sides of the Eq. (1-A) of ξ, substituting $(\xi+i)$ for (ξ) and considering the real part of the equation, $C_1^0(\rho,\xi)$ is obtained.

$$J_1^0(\rho,\xi)=\frac{1}{2}\ln\left(\frac{R^2+R\sqrt{2}\left(\xi\sqrt{1+\frac{\rho^2+\xi^2-1}{R^2}}+\sqrt{1-\frac{\rho^2+\xi^2-1}{R^2}}\right)+\left(1+\xi^2\right)}{\left(\xi+\sqrt{\rho^2+\xi^2}\right)^2}\right) \tag{9-A}$$

Also, $J_0^1(\rho,\xi)$ is calculated by integrating both sides of Eq. (7-A) of ξ; then, by substituting $(\xi+i)$ for (ξ), the real part of equation is considered[23].
