# Supplementary material for: Mechanical properties of mandibular and maxillary bone collagen fibrils based on nonlocal elasticity theory
Source: Biophys Rep (N Y). 2025 Apr 17;5(2):100210. doi: 10.1016/j.bpr.2025.100210 (PMC12138556; doi:10.1016/j.bpr.2025.100210)
Supplement: Document S2. Article plus supporting material [file mmc2.pdf]

Biophysical Reports

ARTICLE

Mechanical properties of mandibular and maxillary bone collagen fibrils based on nonlocal elasticity theory

Elaheh Alibeigi Beni,[1,2,3,*] Alireza Shahidi,[1,2,3] and Behnaz Ebadian[2]
[1]Department of Mechanical Engineering, Isfahan University of Technology, Isfahan 84156-83111, Iran; [2]Department of Prosthodontics, Dental Implants Research Center, Dental Research Institute, School of Dentistry, Isfahan University of Medical Sciences, Isfahan, Iran; and [3]Legal Medicine Research Center, Legal Medicine Organization, Tehran, Iran

ABSTRACT In this paper, the mechanical properties of collagen fibrils in the cortical bone and cortical-trabecular bone interface of the human mandible and maxilla have been investigated. Force-indentation curves on wet collagen fibrils are taken by applying the atomic force microscopy nanoindentation technique, and the elastic modulus is measured. The distribution of stress and strain is determined by considering an elastic medium when it is deformed by a rigid cone. Afterward, by applying the nonlocal elasticity theory and the indentation parameters, the nonlocal parameter of the collagen fibrils is calculated at the nanoscale. Finally, the elastic modulus and nonlocal modulus of the collagen fibrils are compared. According to the results, the highest and lowest values of the elastic modulus of the collagen fibrils are determined in the maxillary cortical-trabecular bone interface (4.16 $\pm$ 0.18 MPa) and mandibular cortical bone (2.03 $\pm$ 0.14 MPa), respectively. In general, in collagen fibrils, this parameter is higher in the maxillary bone than in the mandibular one. In the upper and lower jaws, the elastic modulus of collagen fibrils in the cortical-trabecular bone interface is higher than that of the cortical bone. In mandibular and maxillary bone collagen fibrils, the range of nonlocal parameter and scaling parameter e_0 are computed as (0.430 $\pm$ 0.013–0.483 $\pm$ 0.011 nm) and (0.269 $\pm$ 0.006–0.302 $\pm$ 0.006), respectively. Also, the highest value of this parameter is recorded in the maxillary cortical-trabecular bone interface. The difference between the nanoscale modulus of collagen fibrils and the elastic modulus at large length scales is significant.

WHY IT MATTERS In this paper, the mechanical properties of collagen fibrils in the cortical bone and cortical-trabecular bone interface of the human mandible and maxilla have been investigated. So far, the elastic modulus of collagen fibrils has been calculated at macroscopic scale, according to the classical (local) continuum theory, whereas the mechanical properties of the bone collagen fibrils, as a nanostructure, are dependent on the size parameter. In this study, nonlocal elasticity theory as modified size-dependent continuum theory is considered to predict the nanostructure correctly. According to the results, the difference between the nanoscale modulus of collagen fibrils and the elastic modulus at large length scales is significant.

INTRODUCTION

Type I collagen, as a structural protein, is the most abundant protein in vertebrates; it is prevalent in organs such as bones, tendon, skin, and the respiratory system (1). Collagen fibrils are responsible for mechanical stability, elasticity, toughness, and strength in tissues. The diameter of a single collagen fibril varies from several tens of nanometers to a micrometer (2). A single collagen fibril is composed of collagen molecules, called tropocollagens. Tropocollagens have a diameter of approximately 1.5 nm and a length of 300 nm; they are staggered in the axial direction of collagen fibrils (3–5). Each tropocollagen is made up of a triple helix of left-handed polypeptide chains twisted together into a right-handed triple helix. Each chain contains amino acid residues (6). The general formula for amino acid sequence of collagen is Gly-Pro-X or Gly-X-Hyp, where Pro is a proline and Hyp is a hydroxyproline residue. X can be various other amino acid residues. Glycine is the

Submitted December 26, 2024, and accepted for publication April 15, 2025.

*Correspondence: elalibeigi@me.iut.ac.ir

Editor: Jorg Enderlein.

https://doi.org/10.1016/j.bpr.2025.100210

© 2025 The Author(s). Published by Elsevier Inc. on behalf of Biophysical Society.
This is an open access article under the CC BY license (http://creativecommons.org/licenses/by/4.0/).

smallest amino acid without a side chain; it is located in the center of the coiled peptide chain (7,8). Type I collagen fibrils have characteristic periodic patterns of 60–70 nm (D-banding). This periodicity leads to the appearance of the regions called gap (~0.6 D) and overlap (~0.4 D) (2,9).

Atomic force microscopy (AFM) nanoindentation has great potential for use in clinical activities; it is recognized as a special technique in the characterization of biological samples at the nanoscale (10). In particular, as a new method, early and accurate diagnosis of cancer and osteoporosis has become possible through nanomechanical characterization of individual collagen fibrils (10–12). In addition, the correlation of collagen mutation with some diseases such as osteogenesis, imperfecta, and osteoporosis has been investigated (13,14). Thus, during the last two decades, significant scientific attention has been given to the measurement of the elastic modulus of individual collagen fibrils by applying the AFM nanoindentation technique (10).

At the nanoscale, the dimensions of a system are comparable to the intermolecular and interatomic space of the system. For this reason, the structure cannot be modeled as a continuous medium anymore. Also, at very small scales, the physical properties of the materials change due to the significant effect of intermolecular and interatomic cohesive forces, as well as the discrete nature of the structure. These effects are referred to as the "size" effect (15). Therefore, the mechanical properties of nanostructures such as collagen fibrils are dependent on the size parameter (16). The classical continuum theory has the potential for the analysis of macroscopic structures and is not capable of accounting for the size parameter of nanostructures. Considering the deficiency of classical continuous theories in applying the size effects, using higher-order continuous theories, which can lead to obtaining accurate results by considering size effects, has been highly recommended (17). In this relation, Eringen (18) introduced nonlocal elasticity theory to incorporate nanoscale effects in the classical continuum theory.

Investigating the structure and mechanical behavior of jaw bones is, therefore, important since it leads to the improvement of the function and design of implants. It also minimizes crestal resorption and the possibility of bone-implant junction failure (19). Although the mechanical properties of mineralized fibrils in the jaw bones at the submicron scale have been reported in the literature (20,21), the elastic properties and topographic features of collagen fibrils in the cortical bone and cortical-trabecular bone interface are not available. Also, at the nanoscale, the mechanical properties of collagen fibrils, by employing higher-order continuous theories and indentation parameters, have not been investigated. Furthermore, the difference between the nanoscale properties of collagen fibrils is not known by considering the small-scale effect and elastic properties at the large length scale.

Therefore, in the present paper, attempts have been made, for the first time, to determine the mechanical properties of collagen fibrils in the cortical bone and cortical-trabecular bone interface of the human mandible and maxilla by using the AFM indentation method. The elastic modulus of collagen fibrils is calculated using the Oliver-Pharr method (22). The distribution of stress and strain is determined by considering a semi-infinite elastic solid when its plane surface is deformed under the pressure of a rigid cone (23). By considering the stress and strain tensor components and indentation parameters and by employing the size-dependent nonlocal elasticity theory, the nonlocal parameter is calculated at the nanoscale. Finally, the values of elastic modulus, nonlocal modulus, and the scaling parameter, e_0, in mandibular and maxillary collagen fibrils are compared.

MATERIALS AND METHODS

Specimen preparation

The specimens of mandibular and maxillary bones were obtained from the fresh cadaver of male donors (21 and 37 years old) at autopsy (at Shahrekord Legal Medicine Organization, ethics committee's approval was obtained, approval no. IR.UIT.REC.1401.001). The times from death to specimen procurement (procurement delay) were 12 and 20 h, respectively. As shown in Fig. 1, bone specimens were harvested from the anterior part of the ramus, from the mandibular second premolars to the distal of the second molars, and the maxillary second premolars to the distal of the second molars regions. Three anatomically characteristic directions, including inferosuperior (Fig. 1, *blue*), buccolingual (Fig. 1, *red*), and mesiodistal ones, were marked. Bone specimens were stored in a solution consisting of 95.5% ethanol and 0.9% normal saline in equal proportions (24). Thereafter, bone slices parallel to the occlusal plane were sectioned using an automatic cutting machine on the mandibular and maxillary bone specimens. In all steps, bone slices were kept in a moist state and exposed to fresh deionized water. In general, nine and eight slices were obtained on the mandibular and maxillary bones, respectively. The cutting speed was 10 μm/s. The thickness of each of the slices was 75–200 μm. Also, the dimensions of each of the slices were approximately 3 $\times$ 5 mm (width $\times$ height). Afterward, in each of the slices, the bone marrow was gently cleaned by a soft brush and water jet.

Collagen fibrils

In the demineralization process, all the slices were soaked in a solution consisting of 22.5% formic acid and sodium citrate solution (100 g/L) in equal proportions. The solution was changed once a day. The decalcification process was completed by verifying the concentration level of free calcium in the decalcifying solution. For this purpose, after each change, 5 mL of decalcifying solution

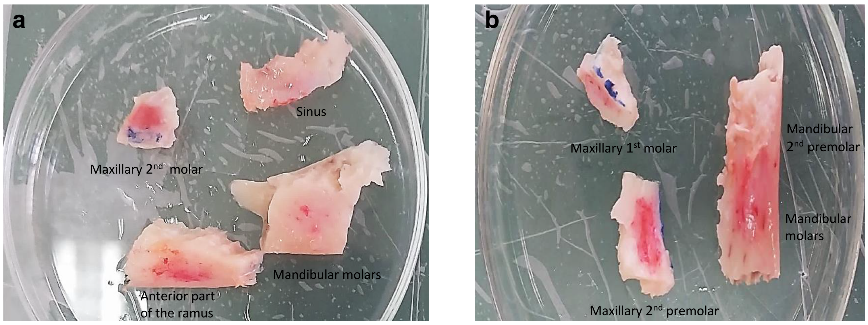

FIG. 1 Different anatomical regions of fresh mandibular and maxillary bone specimens belonging to a 37-year-old-man (*a*) and a 21-year-old-man (*b*). Three anatomically characteristic directions, including inferosuperior (*blue*), buccolingual (*red*), and mesiodistal ones, are marked.

was mixed with 5 mL of 0.5 N sodium hydroxide (NaOH) and 1 mL of 5% ammonium oxalate solution (25,26). As shown in Fig. 2, turbidity in the final mixture indicates the presence of calcium ions. This process was repeated until the final solution became clear. The highest amount of calcium ions was observed on the first day, whereas on the eighth day of decalcification, the mixture was free of calcium ions. The durations of mandibular bone and maxillary bone decalcification were 8–9 and 4–5 days, respectively. After decalcification, to remove traces of acid, the slices were washed five times in a sonic water bath with deionized water. In this step, the temperature of the deionized water did not exceed 30°C. Thereafter, the slices were kept in deionized water at a temperature of 3°C until the AFM nanoindentation test.

AFM nanoindentation tests

AFM (NT-MDT Company) experiments were carried out. For AFM imaging and the nanoindentation test, an AFM tip (CSG10) with a curvature radius of 6 nm and a spring constant of 0.5 N/m was used (27). Contact mode was then performed in the AFM imaging procedure. The nanoindentation tests were conducted on some random positions selected on different collagen fibrils of the occlusal plane and under the ambient state.

In this study, cantilever deflection signals – force-height curves on the different overlap regions were obtained to measure the elastic modulus. A maximum indentation depth of 30 nm and a velocity of the tip in loading and unloading of 1000 nm/s were considered. 81 AFM nanoindentation tests on collagen fibrils in the cortical bone and cortical-trabecular bone interface were also performed. These regions are shown in the mandibular and maxillary bones in Fig. 3. The substrate material of the AFM test was steel (annealing process). The air at room temperature and humidity was 18°C and 45%, respectively. The maximum duration of indentations in each of the jaw bones and each of the cortical bones and cortical-trabecular bone interfaces was 60 min. A three-dimensional AFM image of the maxillary bone collagen fibril is shown in Fig. 4 *A*.

Determination of indentation modulus

According to the Oliver-Pharr method, contact stiffness can be calculated from the unloading part (Fig. 4 *B*) of the force-indentation curve (22). Then, by applying the indenter force, contact depth, elastic modulus of the indenter, Poisson's ratio of the sample, and the indenter, the elastic modulus of collagen fibrils is calculated. The Oliver-Pharr analysis is presented in the supporting material.

Nonlocal elasticity theory at small scales

According to this theory, in the domain, the stress at the reference point is a function of the strains at all other points. $(\alpha = e_0 l_i / l_e)$ is the nonlocal modulus, which represents the scale coefficient or nonlocal parameter of the length unit. In fact, this parameter incorporates the effect of the nanoscale on mechanical behavior. l_i and l_e also stand for internal characteristic lengths and external characteristic lengths, respectively. e_0 is a constant parameter that is appropriate for each material. In other words, this parameter should be determined for each nanostructure independently (18,28). Collagen fibril is assumed to be a homogenous and isotropic solid. The Poisson's ratio is also considered to be 0.5 (29,30). The distribution of stress and strain in a semi-infinite elastic medium, when it is deformed by a rigid conical indenter, is determined (23). Fig. 5 *A* shows the indentation parameters (31). By substituting the contact depth, contact radius, Poisson's ratio, and elastic modulus of the fibrils, the components of the stress and strain tensors are obtained. Afterward, by applying the nonlocal elasticity theory, the nonlocal parameter $(\mu = e_0 l_i)$ of the collagen fibrils is calculated in the elastic

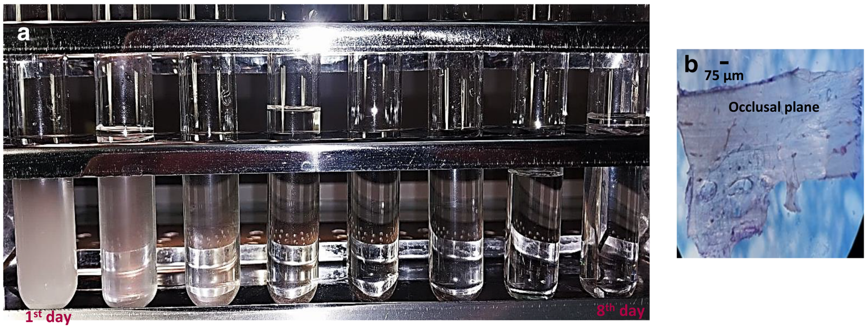

FIG. 2 Demineralization of mandibular bone slices (*a*). Bone slice with the thickness of around 75 μm (*b*). Turbidity in the final mixture indicates the presence of calcium ions. The demineralization process is repeated until the mixture becomes clear. The highest amount of calcium ions is observed on the first day, whereas on the eighth day of decalcification, the mixture is free of calcium ions.

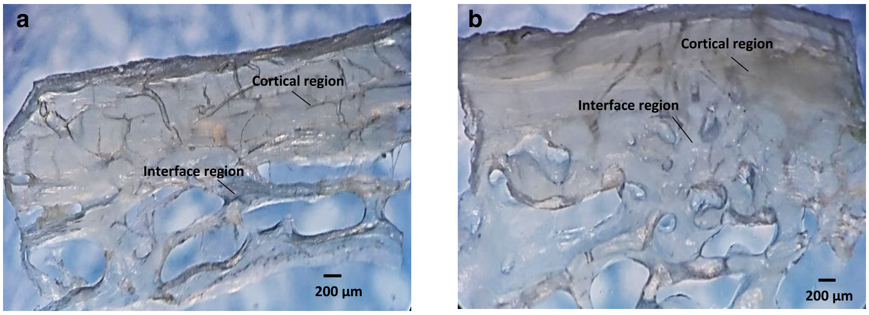

FIG. 3 Demineralized bone slices in mandible (*a*) and maxilla (*b*). The slice thickness is around 200 μm.

medium. Finally, the scaling parameter, e_0, can be obtained at the nanoscale. The components of stress and strain, as well as the nonlocal elasticity theory, are presented in the supporting material.

RESULTS

The values of elastic modulus (mean $\pm$ SD) of the collagen fibrils in mandibular and maxillary bone are shown in Table 1. The value of this parameter varies in cortical bone and the cortical-trabecular bone interface. The elastic moduli measured in the mandibular and maxillary bones were in the range of 2.03 $\pm$ 0.14–2.78 $\pm$ 0.20 and 3.08 $\pm$ 0.04–4.16 $\pm$ 0.18 MPa, respectively. According to the results, the elastic modulus of collagen fibrils in the cortical-trabecular bone interface was higher than that of the cortical one. Also, this parameter in collagen fibrils in cortical as well as cortical-trabecular interface regions of the maxillary bone was higher than that of the mandibular bone.

There was no significant difference in the D-periodic banding pattern between mandibular and maxillary collagen fibrils. The diameter of individual collagen fibrils varied from 159 to 278 nm; additionally, the gap-overlap periodicity was close to 67 nm. Also, it seems that in the upper and lower jaw bones, no association was detected between the diameter of collagen fibrils and their elastic modulus (32). Table 2 presents the values (mean $\pm$ SD) of nonlocal parameters, as well as the parameter e_0 in the collagen fibrils.

The value of μ in the mandibular and maxillary bones varied from 0.430 $\pm$ 0.013 to 0.461 $\pm$ 0.006 and 0.453 $\pm$ 0.004 to 0.483 $\pm$ 0.011 nm, respectively. The highest and lowest values of the nonlocal parameter in collagen fibrils were recorded in the maxillary cortical-trabecular bone interface and mandibular cortical bone, respectively. The difference in the values of the nonlocal parameter in both jaw bones can be attributed to the difference in the values of stiffness and contact depth between the tip and collagen fibrils. The diameter of a tropocollagen is 1.4–1.6 nm (2). The diameter of a tropocollagen is shown in Fig. 6 *E*. The scaling parameter, e_0, is calculated by assuming that the internal characteristic length is equal to the diameter of a collagen molecule. In the collagen fibrils of maxillary bone, the difference of the constant parameter, e_0, between the cortical bone and the cortical-trabecular bone

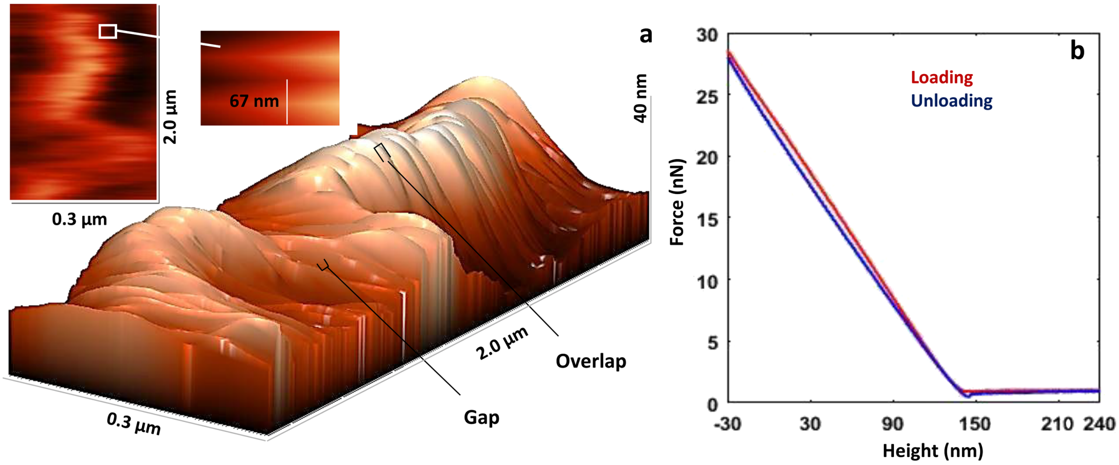

FIG. 4 (*a*) Three-dimensional (3D) AFM image showing D-periodic banding pattern of the maxillary bone collagen fibril. 2D top-view topography AFM images and their banding patterns (67 nm) and (*b*) force (nN)- height (nm) curve.

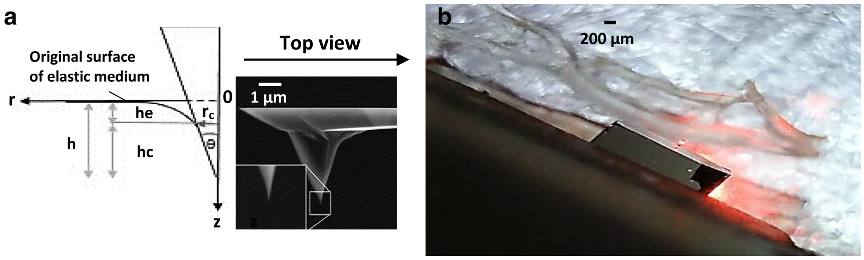

FIG. 5 (*a*) A schematic representation of a conic tip indenting a half-space solid medium (*left*). The side view of the AFM tip (CSG10) is shown (*right*). Indentation parameters contain the maximum displacement of the tip (h), contact depth (h_c), elastic displacement of the solid surface at the contact line with the tip (h_e), cone angle (θ), and contact radius (r_c). (*b*) The top view of the AFM cantilever during the nanoindentation test on the mandibular collagen fibrils.

interface is 3.57%. In the maxillary cortical-trabecular bone interface, the ratio of the elastic modulus to the nonlocal modulus is approximately 8.5.

Table 3 presents a rigorous statistical analysis, including tests for the significance of differences in the elastic modulus and nonlocal parameters.

DISCUSSION

In the present study, the elastic modulus, nonlocal parameter, and constant parameter, e_0, of the collagen fibrils of human mandibular and maxillary bones were obtained. In both the upper and lower jaw bones, at least nine nanoindentation tests were performed on the collagen fibrils in each of the cortical and cortical-trabecular interface regions. More nanoindentation tests were avoided due to the decrease in the humidity of the fibrils, change in the elastic properties, and the reduction of the accuracy of the measurement (33). Force-indentation curves on the overlap regions, on the central line, and in the areas parallel to the long axis of the collagen fibrils were taken. The number of force-displacement curves is equal to the number of nanoindentations. The top view of the AFM cantilever during the nanoindentation test on the mandibular collagen fibrils is presented in Fig. 5 *B*. The gap region was softer and more deformable than the overlap region. According to a study conducted on bovine Achilles tendon collagen fibrils, the elastic modulus varied in gap and overlap regions (2). In the gap region, due to the increase of the contact area between the collagen fibril and the tip, the stiffness of the fibrils was overestimated (33).

Cortical bone is denser than cancellous bone. The properties of the upper and lower jaws at the submicron scale were examined in one study. Based on the results, the elastic modulus of the cortical bone was reported to be higher than that of the trabecular one. Also, the value of this parameter was higher in the mandibular bone than in the maxillary one (20). Meanwhile, at the nanoscale, in both the upper and lower jaw bones, the elastic modulus of collagen fibrils in the cortical-trabecular interface region was higher than that of the cortical one. The static elastic modulus of mineralized fibrils is directly related to the amount of minerals deposited in the collagen matrix. Specifically, this parameter is increased with raising mineral density (34,35). In addition, the mechanical properties of mineralized tissue depend on the arrangement of the crystals (36).

To preserve tissue and retain the mechanical properties of collagen fibrils, a weak acid was used in the demineralization process. For this reason, acid etching was time consuming (26). Also, the maxillary bone demineralized faster than the mandibular bone. The mineral form of bone is divided into interfibrillar and extrafibrillar parts. The interfibrillar part, which is located in the gap zone between collagen molecules, consists of 25%–30% mineral material. Approximately 70%–75% of the mineral form is etched rapidly, whereas the other remaining mineral is removed at a slow rate (37,38). The characteristic 60–70 nm periodicity of the gap region did not appear

TABLE 1 The elastic modulus values of the collagen fibrils in the mandibular and maxillary bone

Age/sex	Bone		No. of fibrils	No. of indentations	No. of locations per fibril	Elastic modulus (MPa) ± SD
37-year-old male	mandible	cortical bone	2	10	5, 5	2.03 ± 0.14
		cortical-trabecular bone interface	2	9	5, 4	2.15 ± 0.05
	maxilla	cortical bone	3	12	4, 5, 3	3.74 ± 0.44
		cortical-trabecular bone interface	3	10	3, 4, 3	4.16 ± 0.18
21-year-old male	mandible	cortical bone	3	12	4, 4, 4	2.51 ± 0.36
		cortical-trabecular bone interface	3	10	3, 4, 3	2.78 ± 0.20
	maxilla	cortical bone	2	9	4, 5	3.08 ± 0.04
		cortical-trabecular bone interface	2	9	4, 5	3.55 ± 0.03

TABLE 2 The values of the nonlocal parameter and scaling parameter, e_0, of collagen fibrils in the mandibular and maxillary bone

	Mandibular cortical bone		Mandibular cortical-trabecular bone interface		Maxillary cortical bone		Maxillary cortical-trabecular bone interface	
Age/sex	μ(nm)	e_0	μ(nm)	e_0	μ(nm)	e_0	μ(nm)	e_0
37-year-old male	0.438 ± 0.017	0.274 ± 0.006	0.430 ± 0.013	0.269 ± 0.006	0.471 ± 0.011	0.294 ± 0.006	0.483 ± 0.011	0.302 ± 0.006
21-year-old male	0.459 ± 0.006	0.287 ± 0.003	0.461 ± 0.006	0.288 ± 0.003	0.453 ± 0.004	0.283 ± 0.001	0.464 ± 0.005	0.290 ± 0.002

on mineralized fibrils, but it was completely revealed with continuous decalcification (29). Therefore, completing the demineralization process of collagen fibrils is strongly recommended because it leads to a more accurate measurement of elastic modulus by applying the AFM nanoindentation technique.

The maximum indentation depth depends on the height of the collagen fibrils and the curvature radius of the tip. In the AFM technique, to avoid the influence of the substrate and underlying and neighboring collagen fibrils on the measurements, the maximum indentation depth is considered small enough. Therefore, the maximum indentation depth should be lower than 5%–15% of the diameter of the fibrils (1,2,33). Considering this range of indentation depth in the force-indentation curve, as well as applying a maximum indentation force lower than 30 nN, the mechanical deformation in collagen fibrils is elastic (2). In the present study, the elastic modulus of collagen fibrils was not changed at loading rates up to 1000 nm/s. In one study, the static nanoindentation measurement was conducted on the collagen fibers of bovine Achilles tendon, showing that the elastic modulus did not change for tip velocities up to 1500 nm/s (6). Employing a relatively fast loading rate and a consistent loading-unloading rate can prevent the potential effects of viscoelasticity on the static nanoindentation measurements (25,32).

Bone specimens have been primarily harvested from older subjects because these specimens are available in donors' cadavers (39). There are limitations to obtaining fresh samples of young donors' cadavers. Also, available maxillary cortical bone is weak and limited in quantity; preparing the specimen and

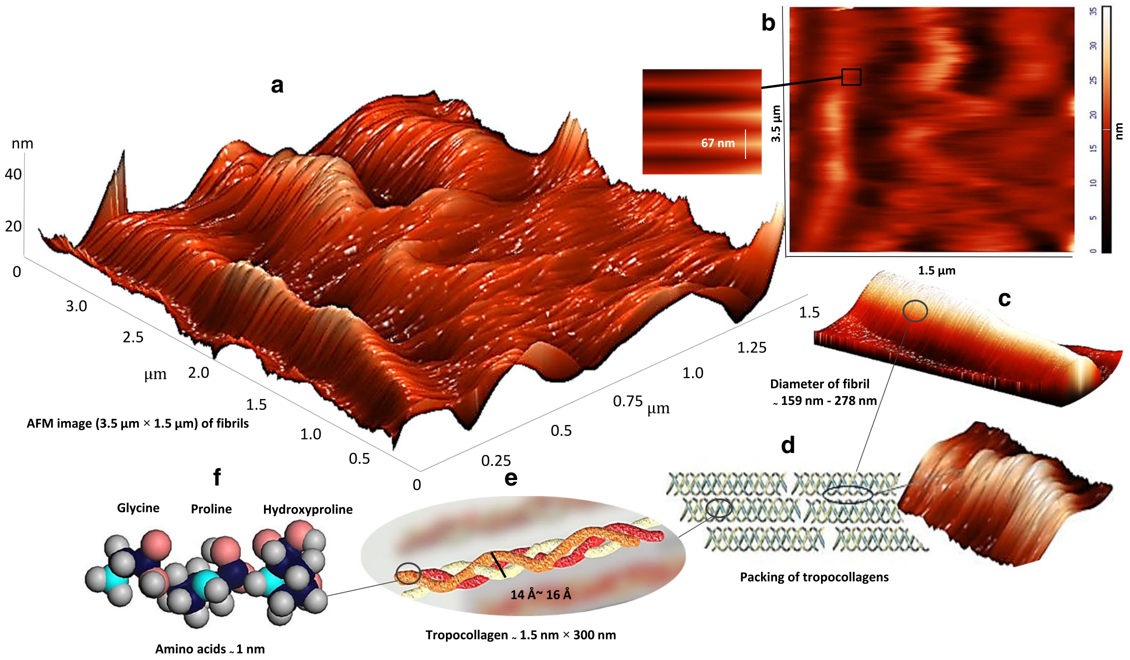

FIG. 6 The structural hierarchy of type I collagen fibrils of mandibular cortical bone. Different stages of fibrillogenesis are illustrated from the primary structure at the nanoscale to fibrils at the submicron scale. (*a*) Three-dimensional AFM image (3.5 × 1.5 μm) was obtained of collagen fibrils presenting the characteristic banding pattern. (*b*) Two-dimensional AFM image (right) presenting collagen fibrils and showing the D-periodic banding pattern (67 nm) of the collagen fibril. (*c*) The range of type I collagen fibril diameters is shown. (*d*) The collagen fibril is composed of a packing of tropocollagens. (*e*) A tropocollagen is made up of polypeptide chains. (*f*) Each chain contains amino acid residues.

TABLE 3 Statistically significant (p < 0.05) results of t-test for differences in elastic modulus and nonlocal parameter

Age/sex	Bone	p value of elastic modulus	Bone	p value of nonlocal parameter
37-year-old male	Mac1 < Mai1	0.043	Mac1 = Mai1	0.343
	Mxc1 < Mxi1	0.034	Mxc1 = Mxi1	0.069
	Mac1 < Mxc1	0.001	Mac1 < Mxc1	<0.001
	Mai1 < Mxi1	< 0.001	Mai1 < Mxi1	<0.001
21-year-old male	Mac2 < Mai2	0.039	Mac2 = Mai2	0.607
	Mxc2 < Mxi2	0.048	Mxc2 < Mxi2	0.001
	Mac2 < Mxc2	0.012	Mac2 = Mxc2	0.162
	Mai2 < Mxi2	0.013	Mai2 = Mxi2	0.288

Donor 1 is a 37-year-old male, and donor 2 is a 21-year-old male. For example, Mac1 is the mandibular cortical of a 37-year-old male. Mac, mandibular cortical; Mai, mandibular cortical-trabecular interface; Mxc, maxillary cortical; Mxi, maxillary cortical-trabecular interface.

measuring the mechanical properties of this bone are difficult. So far, in a few previous studies, the effect of aging on the elastic properties of collagen fibrils has been reported. The mechanical properties of individual type II collagen fibrils of articular cartilage have been investigated using the nanoindentation test, and variations of the elastic modulus with increasing age and osteoarthritis process have been obtained (40). In the present study, predicting the aging effect on elastic properties could be difficult. According to the results, in the mandibular bone, the elastic modulus of collagen fibrils in the cortical bone and cortical-trabecular bone interface decreased with age. Meanwhile, in maxillary bone collagen fibrils, elastic modulus values increased with age. Therefore, to investigate the aging effect on the mechanical properties of collagen fibrils, future studies with more samples should be planned.

So far, in many studies, the elastic modulus of native collagen fibers has been calculated using the AFM nanoindentation technique at the dehydrated state. In this regard, an elastic modulus of fibrils extracted from sea cucumber of 1–2 GPa was measured (41). In other studies conducted on individual type I collagen fibrils from rat tail tendon, the static elastic moduli were reported to be 3.2 ± 1.1 (1), 5–11.5 (33), and 1–10 (42) GPa. In wild mouse tail tendon fibrils, a value of 7.0 ± 1.5 GPa was recorded (1). Meanwhile, in other studies conducted on bovine Achilles tendon collagen fibrils, 1.9 ± 0.5 (43), 1.2–2.2 (2), 0.5 (44), and 0.9 ± 0.16–1.16 ± 0.27 (45) GPa were reported. Meanwhile, under the hydrated state, the value of the elastic modulus of fibrils prepared from human dentin was in the range of 30–60 MPa (29). This parameter was measured to be 172.5 ± 59 (6) and 5–10 (46) MPa in the individual collagen fibrils from bovine Achilles tendon and porcine sclera, respectively. According to the range of elastic modulus values determined in the literature, the importance of hydration has been highlighted. Collagen fibrils have interstitial water, and drying the fibers increases their stiffness (33). The elastic modulus values in the present study were also in the same order of magnitude as the data obtained under the hydrated state.

In the previous studies, the elastic modulus of collagen fibrils has been calculated at large length scales (1,29). According to the classical (local) continuum theory, in the macroscopic structure, the stress at a point is assumed to be a function of the strain at that point (28). The mechanical properties of the bone collagen fibrils as a nanostructure are dependent on the size parameter (16). In this study, nonlocal elasticity theory as modified size-dependent continuum theory is considered to predict the nano structure correctly. Therefore, at the reference point of the domain, the stress depends not only on the strains at that point but also on the strains at all other points of the domain. In size-dependent nonlocal elasticity theory, a nonlocal modulus indicates small-scale effect intensity (15). The key parameter in this theory at the nanoscale is the constant parameter, e_0. However, there have been few rigorous studies on the estimation of this parameter for different physical problems. The constant parameter, e_0, was proposed by Eringen (47) to be 0.39 in a single-walled carbon nanotube. According to the results, in mandibular and maxillary bone collagen fibrils, there was a significant difference between the elastic modulus at large length scales and the nonlocal modulus at the nanoscale. In the lower jaw, there was no significant difference between the constant parameter, e_0, in the cortical bone and the cortical-trabecular bone interface. Also, there was no significant association between this parameter and the age of subjects.

CONCLUSION

In this paper, the mechanical properties and topographical features of the collagen fibrils of mandibular and maxillary bones were investigated at the nanoscale. The elastic modulus of wet collagen fibrils in the cortical bone and cortical-trabecular bone interface was calculated using the AFM indentation method, force-indentation data, and the local continuum theory. At the nanoscale, the nonlocal parameter was computed by applying the nonlocal elasticity theory and the components of the stress and strain tensor. No significant difference was observed between the upper and lower jaw bones in the topography of collagen fibrils. Meanwhile, there were differences in the mechanical properties of mandibular and

maxillary bones. The elastic modulus of maxillary bone fibrils was higher than that of the mandibular bone. Also, this parameter was found to be more in the cortical-trabecular bone interface than the cortical bone. The range of scaling parameter, e_0, in mandibular and maxillary bones was 0.269–0.302.

ACKNOWLEDGMENTS

The authors would like to thank the Legal Medicine center of Chaharmahal and Bakhtiari, Shahrekord, Iran. As well as, Dr. Alireza Tamizifar of the Shahrekord University of Medical Science for providing bone samples.

AUTHOR CONTRIBUTIONS

E.A. conceived and designed the analysis and analysis tool, performed the analysis, wrote the paper, and provided other contributions. A.S. contributed to the design and analysis tool of the work, performed the text mining analysis, and provided supervision, investigation, and final approval of the version to be published. B.E. obtained the samples, performed the text mining analysis, and provided final approval of the version to be published.

DECLARATION OF INTERESTS

The authors have declared that no conflict of interest exists.

SUPPORTING MATERIAL

Supplemental information can be found online at https://doi.org/10.1016/j.bpr.2025.100210.

REFERENCES

1. Andriotis, O. G., W. Manuyakorn, …, P. J. Thurner. 2014. Nanomechanical assessment of human and murine collagen fibrils via atomic force microscopy cantilever-based nanoindentation. *J. Mech. Behav. Biomed. Mater.* 39:9–26.
2. Minary-Jolandan, M., and M.-F. Yu. 2009. Nanomechanical heterogeneity in the gap and overlap regions of type I collagen fibrils with implications for bone heterogeneity. *Biomacromolecules.* 10:2565–2570.
3. Hay, E. D. 1991. Cell biology of extracellular matrix. Springer Science & Business Media.
4. Hulmes, D. J., A. Miller, …, J. Woodhead-Galloway. 1973. Analysis of the primary structure of collagen for the origins of molecular packing. *J. Mol. Biol.* 79:137–148.
5. Orgel, J. P., A. Miller, …, T. J. Wess. 2001. The in situ supermolecular structure of type I collagen. *Structure.* 9:1061–1069.
6. Grant, C. A., D. J. Brockwell, …, N. H. Thomson. 2009. Tuning the elastic modulus of hydrated collagen fibrils. *Biophys. J.* 97:2985–2992.
7. Ottani, V., D. Martini, …, M. Raspanti. 2002. Hierarchical structures in fibrillar collagens. *Micron.* 33:587–596.
8. Szpak, P. 2011. Fish bone chemistry and ultrastructure: implications for taphonomy and stable isotope analysis. *J. Archaeol. Sci.* 38:3358–3372.
9. Baniasadi, M., and M. Minary-Jolandan. 2015. Alginate-collagen fibril composite hydrogel. *Materials.* 8:799–814.
10. Kontomaris, S. V., A. Stylianou, and A. Malamou. 2022. Atomic force microscopy nanoindentation method on collagen fibrils. *Materials.* 15:2477.
11. Plodinec, M., M. Loparic, …, C. A. Schoenenberger. 2012. The nanomechanical signature of breast cancer. *Nat. Nanotechnol.* 7:757–765.
12. Stolz, M., R. Gottardi, …, U. Aebi. 2009. Early detection of aging cartilage and osteoarthritis in mice and patient samples using atomic force microscopy. *Nat. Nanotechnol.* 4:186–192.
13. Layton, B. E., A. M. Sastry, …, M. A. Philbert. 2004. Differences between collagen morphologies, properties and distribution in diabetic and normal biobreeding and Sprague–Dawley rat sciatic nerves. *J. Biomech.* 37:879–888.
14. Calò, A., Y. Romin, …, K. Manova-Todorova. 2020. Spatial mapping of the collagen distribution in human and mouse tissues by force volume atomic force microscopy. *Sci. Rep.* 10:15664.
15. Shahidi, A. R., A. Anjomshoa, …, M. Kamrani. 2013. Fundamental size dependent natural frequencies of non-uniform orthotropic nano scaled plates using nonlocal variational principle and finite element method. *Appl. Math. Model.* 37:7047–7061.
16. Ghavanloo, E. 2017. Persistence length of collagen molecules based on nonlocal viscoelastic model. *J. Biol. Phys.* 43:525–534.
17. Alibeigi, B., Y. Tadi Beni, and F. Mehralian. 2018. On the thermal buckling of magneto-electro-elastic piezoelectric nanobeams. *Eur. Phys. J. Plus.* 133:133.
18. Eringen, A. C. 1983. On differential equations of nonlocal elasticity and solutions of screw dislocation and surface waves. *J. Appl. Phys.* 54:4703–4710.
19. Misch, C. E., Z. Qu, and M. W. Bidez. 1999. Mechanical properties of trabecular bone in the human mandible: implications for dental implant treatment planning and surgical placement. *J. Oral Maxillofac. Surg.* 57:700–708.
20. Seong, W.-J., U.-K. Kim, …, C.-C. Ko. 2009. Elastic properties and apparent density of human edentulous maxilla and mandible. *Int. J. Oral Maxillofac. Surg.* 38:1088–1093.
21. Kim, D.-G., Y.-H. Jeong, …, W. M. Johnston. 2015. Regional variation of bone tissue properties at the human mandibular condyle. *Bone.* 77:98–106.
22. Oliver, W. C., and G. M. Pharr. 2004. Measurement of hardness and elastic modulus by instrumented indentation: Advances in understanding and refinements to methodology. *J. Mater. Res.* 19:3–20.
23. 1948. Boussinesq's problem for a rigid cone. *In* Mathematical Proceedings of the Cambridge Philosophical Society. I. N. Sneddon, ed Cambridge University Press.
24. Ashman, R. B., S. C. Cowin, …, J. C. Rice. 1984. A continuous wave technique for the measurement of the elastic properties of cortical bone. *J. Biomech.* 17:349–361.
25. Leng, H., M. J. Reyes, …, X. Wang. 2013. Effect of age on mechanical properties of the collagen phase in different orientations of human cortical bone. *Bone.* 55:288–291.
26. Yeni, Y. N., M. B. Schaffler, …, D. P. Fyhrie. 2002. Prestress due to dimensional changes caused by demineralization: a potential mechanism for microcracking in bone. *Ann. Biomed. Eng.* 30:217–225.
27. https://www.ntmdt-tips.com/products/view/csg10.
28. Babaei, H., and A. R. Shahidi. 2011. Small-scale effects on the buckling of quadrilateral nanoplates based on nonlocal elasticity theory using the Galerkin method. *Arch. Appl. Mech.* 81:1051–1062.
29. Balooch, M., S. Habelitz, …, G. W. Marshall. 2008. Mechanical properties of mineralized collagen fibrils as influenced by demineralization. *J. Struct. Biol.* 162:404–410.

30. Mott, P. H., J. R. Dorgan, and C. m. Roland. 2008. The bulk modulus and Poisson's ratio of "incompressible" materials. *J. Sound Vib.* 312:572–575.

31. Sirghi, L., and F. Rossi. 2006. Adhesion and elasticity in nanoscale indentation. *Appl. Phys. Lett.* 89:243118.

32. Tang, B., M. K. Fong, …, W. W. Lu. 2014. Nanostiffness of collagen fibrils extracted from osteoarthritic cartilage characterized with AFM nanoindentation. *Soft Mater.* 12:253–261.

33. Wenger, M. P. E., L. Bozec, …, P. Mesquida. 2007. Mechanical properties of collagen fibrils. *Biophys. J.* 93:1255–1263.

34. Currey, J. D. 1969. The relationship between the stiffness and the mineral content of bone. *J. Biomech.* 2:477–480.

35. Currey, J. D. 1990. Physical characteristics affecting the tensile failure properties of compact bone. *J. Biomech.* 23:837–844.

36. Weiner, S., and H. D. Wagner. 1998. The material bone: structure-mechanical function relations. *Annu. Rev. Mater. Sci.* 28:271–298.

37. Bonar, L. C., S. Lees, and H. A. Mook. 1985. Neutron diffraction studies of collagen in fully mineralized bone. *J. Mol. Biol.* 181:265–270.

38. Pidaparti, R. M., A. Chandran, …, C. H. Turner. 1996. Bone mineral lies mainly outside collagen fibrils: predictions of a composite model for osternal bone. *J. Biomech.* 29:909–916.

39. Dechow, P. C., G. A. Nail, …, R. B. Ashman. 1993. Elastic properties of human supraorbital and mandibular bone. *Am. J. Phys. Anthropol.* 90:291–306.

40. Wen, C.-Y., C.-B. Wu, …, K. Y. Chiu. 2012. Collagen fibril stiffening in osteoarthritic cartilage of human beings revealed by atomic force microscopy. *Osteoarthr. Cartil.* 20:916–922.

41. Heim, A. J., W. G. Matthews, and T. J. Koob. 2006. Determination of the elastic modulus of native collagen fibrils via radial indentation. *Appl. Phys. Lett.* 89:181902.

42. Andriotis, O. G., K. Elsayad, …, P. J. Thurner. 2019. Hydration and nanomechanical changes in collagen fibrils bearing advanced glycation end-products. *Biomed. Opt. Express.* 10:1841–1855.

43. Grant, C. A., D. J. Brockwell, N. H. Thomson…, 2008. Effects of hydration on the mechanical response of individual collagen fibrils. *Appl. Phys. Lett.* 92:233902.

44. 2012. Mechanical properties of collagen fibrils on thin films by Atomic Force Microscopy nanoindentation. *In* 2012 IEEE 12th International Conference on Bioinformatics & Bioengineering (BIBE). S. Kontomaris, A. Stylianou, and …, K. Politopouloseds IEEE.

45. Kontomaris, S. V., D. Yova, …, G. Balogiannis. 2015. The effects of UV irradiation on collagen D-band revealed by atomic force microscopy. *Scanning.* 37:101–111.

46. Papi, M., P. Paoletti, R. Akhtar…, 2014. Nanoscale characterization of the biomechanical properties of collagen fibrils in the sclera. *Appl. Phys. Lett.* 104:103703.

47. Duan, W. H., C. M. Wang, and Y. Y. Zhang. 2007. Calibration of nonlocal scaling effect parameter for free vibration of carbon nanotubes by molecular dynamics. *J. Appl. Phys.* 101:024305.

Biophysical Reports, Volume *5*

Supplemental information

Mechanical properties of mandibular and maxillary bone collagen fibrils based on nonlocal elasticity theory

Elaheh Alibeigi Beni, Alireza Shahidi, and Behnaz Ebadian

Determination of indentation modulus

According to the Oliver-Pharr method, contact stiffness can be calculated from the unloading part (Fig. 4b) of the force-indentation curve. In this regard, the following equation is fitted on the unloading force–indentation data[22].

$$P = a\left(h - h_f\right)^m \quad (1)$$

, where P is the indenter force. Also, h, h_f stand for the indentation and final indentation depth, respectively; a and m are the fitting parameters as well. h_c represents the contact depth as determined by the following equation.

$$h_c = h_{\max} - \varepsilon \frac{P_{\max}}{S_c} \quad (2)$$

, where the contact stiffness, S_c, is the slope of the unloading curve at the maximum indentation depth. In this equation, ε is a constant parameter and a function of the exponent m that depends on the geometry of the tip[1].

$$\varepsilon(m) = m\left[1 - \frac{2\Gamma(m/2(m-1))}{\sqrt{\pi}\,\Gamma(1/2(m-1))}(m-1)\right] \quad (3)$$

In the forgoing equation, Γ stands for Gamma function. The reduced modulus from indentation experiments is calculated according to the following equation [22].

$$E_r = \frac{\sqrt{\pi} S_c}{2\sqrt{A_c}} \quad (4)$$

Fig. 5. shows the indentation parameters[31].

The contact area is determined as follows[31]:

$$A_c = \pi \tan^2(\theta) h_c^2 \quad (5)$$

The reduced modulus is a combination of E_{sample}, the elastic modulus of the sample and $E_{indenter}$ the elastic modulus of the indenter.,

$$\frac{1}{E_r} = \frac{1 - \nu_{sample}^2}{E_{sample}} + \frac{1 - \nu_{indenter}^2}{E_{indenter}} \quad (6)$$

In this equation, ν_{sample}, $\nu_{indenter}$ stand for the Poisson's ratio of the sample and indenter, respectively. In the case of, the tip is much stiffer than the sample, and the second term of Eq. (6) is close to zero. Therefore, the equation is rewritten as follows:

$$\frac{1}{E_r} = \frac{1 - \nu_{sample}^2}{E_{sample}} \quad (7)$$

The distribution of stress and strain

The distribution of stress and strain in a semi-infinite elastic medium, when it is deformed by a rigid conical indenter, is determined. According to Fig. 5. the position of a point in the medium is described by cylindrical polar coordinates (r, θ, z). Due to the symmetry around the z-axis, the

displacement vector is considered as the form $(u_r, 0, u_z)$. Here, u_r and u_z represent the displacement along the r-axis and z-axis, respectively[23].
In addition, $\sigma_r, \sigma_\theta, \sigma_z, \tau_{rz}$ stand for the components of the stress tensor. It should be noted that other components of the stress tensor, $\tau_{r\theta}, \tau_{z\theta}$, are equal to zero at any point of the solid.
Considering the equations of elastic equilibrium, it is as follows:

$$\begin{aligned} &\frac{\partial \sigma_r}{\partial r} + \frac{\partial \tau_{rz}}{\partial z} + \frac{1}{r}(\sigma_r - \sigma_\theta) = 0 \\ &\frac{\partial \tau_{rz}}{\partial r} + \frac{\partial \sigma_z}{\partial z} + \frac{\tau_{rz}}{r} = 0 \end{aligned} \qquad (8)$$

Over the entire surface $z = 0$, the shear stress equals zero. The boundary conditions are assumed to be as follows:

$$\begin{aligned} &[\sigma_z(r)]_{z=0} = 0 \qquad (z = 0, \quad r > r_c) \\ &[\tau_{rz}(r)]_{z=0} = 0 \qquad (z = 0, \quad r \geq 0) \end{aligned} \qquad (9)$$

Eqs. (10-15) can satisfy the equations of elastic equilibrium and boundary conditions[23].

$$\frac{r_c \sigma_z}{h_c E} = -\frac{1}{2(1-\nu^2)}\left[J_1^0(\rho,\xi) + \xi\, J_2^0(\rho,\xi)\right] \qquad (10)$$

$$\frac{r_c \tau_{zr}}{h_c E} = -\frac{1}{2(1-\nu^2)}\xi\, J_2^1(\rho,\xi) \qquad (11)$$

$$\frac{r_c \sigma_\theta}{h_c E} = -\frac{1}{2(1-\nu^2)}\left[(2\nu)J_1^0(\rho,\xi) + \frac{1}{\rho}\left[(1-2\nu)J_0^1(\rho,\xi) - \xi J_1^1(\rho,\xi)\right]\right] \qquad (12)$$

$$\frac{r_c(\sigma_r + \sigma_\theta + \sigma_z)}{h_c E} = -\frac{1}{(1-\nu)}J_1^0(\rho,\xi) \qquad (13)$$

$$u_r = h_c\left(\frac{1-2\nu}{2(1-\nu)}J_0^1 - \frac{\xi}{2(1-\nu)}J_1^1\right) \qquad (14)$$

$$u_z = -h_c\left(\frac{\xi}{2(1-\nu)}J_1^0 + J_0^0(\rho,\xi)\right) \qquad (15)$$

In Eqs. (10-13), E and ν represent elastic modulus and Poisson's ratio, respectively. $J_n^m(\rho,\xi)$ is also defined as follows:

$$J_n^m(\rho,\xi) = \int_0^\infty (1-\cos(p))p^{n-2} J_m(\rho p)e^{-p\xi}\,dp \qquad \rho = \frac{r}{r_c}, \quad \xi = \frac{z}{r_c}, \qquad (16)$$

$J_m(\rho p)$ stands for Bessel׳s integral.
As the first step, to determine the distribution of stress and strain along the axis of symmetry, ρ is set to zero.

$$J_1^0(0,\xi)=\int_0^{\infty}\frac{1-\cos p}{p}e^{-p\xi}dp=\int_0^{\infty}\int_0^1\sin(tp)e^{-p\xi}dtdp=\frac{1}{2}\ln\left(1+\frac{1}{\xi^2}\right)$$

$$J_2^0(0,\xi)=\int_0^{\infty}(1-\cos p)e^{-p\xi}dp=\frac{1}{\xi(1+\xi^2)} \tag{17}$$

$$J_0^0(0,\xi)=\int_0^{\infty}\left(\frac{1-\cos p}{p^2}\right)e^{-\xi p}dp=\frac{-\pi}{4\xi}-\frac{\xi}{2}\ln\left(1+\frac{1}{\xi^2}\right)-\xi\left(\ln(p)-\sum_{n=0}^{\infty}\frac{(-\xi p)^n}{n(n!)}\right)_{p=0,\infty}$$

Also, the following result is obtained[23]:

$$\frac{2}{\rho}J_0^1(0,\xi)=J_1^0(0,\xi)$$
$$\frac{2}{\rho}J_1^1(0,\xi)=J_2^0(0,\xi) \tag{18}$$

By substituting Eqs. (17, 18) into Eqs. (10-13), the components of stress on the axis of symmetry are expressed as follows:

$$\frac{r_c\sigma_r}{h_cE}=-\frac{1}{4(1-\nu^2)}\left\{\left(\nu+\frac{1}{2}\right)\ln\left(1+\frac{1}{\xi^2}\right)-\frac{1}{1+\xi^2}\right\}$$
$$\frac{r_c\sigma_z}{h_cE}=-\frac{1}{2(1-\nu^2)}\left\{\frac{1}{2}\ln\left(1+\frac{1}{\xi^2}\right)+\frac{1}{1+\xi^2}\right\} \tag{19}$$
$$\sigma_r=\sigma_\theta$$

Given that $J_2^1(0,\xi)$ is equal to zero τ_{rz} is zero and $\sigma_r,\sigma_z,\sigma_\theta$ in Eq. (19) are the principal stresses at any point of the axis of symmetry. The principal shearing stress, τ , is calculated according to $1/2|\sigma_r-\sigma_z|$. Components of the strain tensor are determined by employing Eqs. (14, 15) and substituting Eq. (17), as follows:

$$\varepsilon_z=\frac{\partial u_z(0,\xi)}{\partial z}=-h_c\left(\left(\frac{1}{4r_c(1-\nu)}-\frac{1}{2r_c}\right)\left(\ln\left(1+\frac{1}{\xi^2}\right)\right)-\frac{1}{2r_c(1-\nu)}\left(\frac{1}{(1+\xi^2)}\right)\right)$$
$$\varepsilon_r=\frac{\partial u_r(0,\xi)}{\partial r}=h_c\left(\frac{1-2\nu}{8r_c(1-\nu)}\ln\left(1+\frac{1}{\xi^2}\right)-\frac{1}{4r_c(1-\nu)}\left(\frac{1}{(1+\xi^2)}\right)\right) \tag{20}$$

ε_{rz} is zero and $\varepsilon_r,\varepsilon_z$ in Eq. (20) are the principal strains at any point of the axis of symmetry. It must be noted that the principal shearing stress along the axis r=0, decreases steadily with increasing (ξ) and it is infinite at the origin of coordinates $\rho=\xi=0$. Therefore, it can be concluded that the distribution of stress and strain at all points of the medium, except the immediate vicinity of the apex of the cone, are obtained by elastic solution. To determine the components of stress and strain tensor at any points in the interior of the elastic medium, the integrals $J_n^m(\rho,\xi)$ are expressed as the following equation[23].

$$J_n^m(\rho,\xi)=Z_n^m(\rho,\xi)-C_n^m(\rho,\xi)\qquad m+n\geq 2 \tag{21}$$

The first integral is evaluated as[23]:

$$Z_n^m(\rho,\xi)=\int_0^\infty p^{n-2}J_m(\rho p)e^{-p\xi}dp=\sum_{k=0}^{\infty}\frac{(-1)^k\left(\frac{1}{2}\rho\right)^{m+2k}}{k!\Gamma(m+k+1)}\int_0^\infty p^{n+m+2k-2}e^{-p\xi}dp$$
$$=\sum_{k=0}^{\infty}\frac{(-1)^k\left(\frac{1}{2}\rho\right)^{m+2k}}{k!\Gamma(m+k+1)}\frac{\Gamma(n+m+2k-1)}{\xi^{n+m+2k-1}} \quad (22)$$

Also, the second integral is defined as:

$$C_n^m(\rho,\xi)=\int_0^\infty p^{n-2}\cos p\, J_m(\rho p)e^{-p\xi}dp \quad (23)$$

By calculating the integrals $J_m^n(\rho,\xi)$ and substituting them into Eqs. (10-13), the components of the stress tensor in the interior of the elastic medium are obtained. The components of the strain tensor are calculated according to the Eq. (24). The details of the integrals $J_m^n(\rho,\xi)$ solution for different values of m and n are presented in Appendix A.

$$\varepsilon_z=\frac{\partial u_z(\rho,\xi)}{\partial z}=-h_c\left(\frac{-J_1^0(\rho,\xi)}{r_c}+\frac{J_1^0(\rho,\xi)}{2r_c(1-\nu)}-\frac{\xi J_2^0(\rho,\xi)}{2r_c(1-\nu)}\right)$$
$$\varepsilon_r=\frac{\partial u_r(\rho,\xi)}{\partial r}=h_c\left(\frac{1-2\nu}{2r_c(1-\nu)}\left(J_1^0(\rho,\xi)-\frac{J_0^1(\rho,\xi)}{\rho}\right)-\left(\frac{\xi}{2r_c(1-\nu)}\right)\left(J_2^0(\rho,\xi)-\frac{J_1^1(\rho,\xi)}{\rho}\right)\right)$$
$$\varepsilon_{rz}=\frac{\partial u_r(\rho,\xi)}{\partial z}+\frac{\partial u_z(\rho,\xi)}{\partial r}=h_c\left(\left(\frac{2\nu-1+\xi r_c}{2r_c(1-\nu)}\right)J_1^1(\rho,\xi)+\frac{\xi}{2r_c(1-\nu)}J_2^1(\rho,\xi)+J_0^1(\rho,\xi)\right) \quad (24)$$
$$\varepsilon_\theta=\frac{u_r(\rho,\xi)}{r}=\frac{h_c}{r}\left(\frac{1-2\nu}{2(1-\nu)}J_0^1(\rho,\xi)-\frac{\xi}{2(1-\nu)}J_1^1(\rho,\xi)\right)$$

Nonlocal elasticity theory at small scales

According to this theory, in the domain, the stress at the reference point is a function of the strains at all other points. The components of the stress tensor for a linear homogenous nonlocal elastic body, neglecting the body force, are expressed according to the following equation.

$$\sigma_{ij}(x)=\int\lambda(|x-x'|,\alpha)C_{ijkl}\varepsilon_{kl}(x')dV(x'),\quad \forall x\in V \quad (25)$$

In the forgoing equation, $\sigma_{ij},\varepsilon_{kl},C_{ijkl}$ represent stress, strain and fourth-order elasticity tensor, respectively. $\lambda(|x'-x|,\alpha)$ is also the nonlocal modulus and has a dimension of $(length)^{-3}$. x stands for a reference point at time t in the body. $|x'-x|$ is the distance in the Euclidean form. $(\alpha=e_0 l_i/l_e)$ represents the scale coefficient or nonlocal parameter of the length unit. In fact, this parameter incorporates the effect of the nano-scale on mechanical behavior. l_i and l_e also

stand for internal characteristic lengths and external characteristic length, respectively. e_0 is a constant parameter that is appropriate for each material. In other words, this parameter should be determined for each nanostructure independently[18, 28]. Due to the difficulty of solving the integral constitutive relation, a differential form equation is used as shown in Eq. (26)[28]:

$$\left(1-\alpha^2 l_e^2 \nabla^2\right)\sigma = C:\varepsilon, \quad \alpha = e_0 l_i / l_e \tag{26}$$

In the forgoing equation, ':' represents the double dot product and $\nabla^2 = \frac{\partial^2}{\partial r^2} + \frac{1}{r}\frac{\partial}{\partial r} + \frac{\partial^2}{\partial z^2}$ refers to the laplacian operator. Collagen fibril is assumed to be a homogenous and isotropic solid. Poisson's ratio is also considered to be 0.5[29]. By considering Eqs. (10-13) and Eq. (24), and substituting the components of stress and strain tensor in Eq. (26) and applying the laplacian operator, nonlocal constitutive relations can be obtained. Finally, the nonlocal parameter $(\mu = e_0 l_i)$ can be obtained in the elastic medium.

Appendix A

In general, $C_m^n(\rho,\xi)$ is calculated by substituting $(\xi + i)$ for (ξ) in Eq. (23); afterward, the real part is equated.

$$Z_2^0(\rho,\xi) = \int_0^\infty e^{-p\xi} J_0(\rho p)dp = \frac{1}{\sqrt{\rho^2+\xi^2}} \tag{1-A}$$

In the following

$$C_2^0(\rho,\xi) = \int_0^\infty \cos p\, e^{-\xi p} J_0(\rho p)dp = \frac{\sqrt{2}}{2R}\sqrt{1+\frac{\rho^2+\xi^2-1}{R^2}} \tag{2-A}$$

$$R^4 = \left(\rho^2+\xi^2-1\right)^2 + 4\xi^2$$

$$J_2^0(\rho,\xi) = \frac{1}{\sqrt{\rho^2+\xi^2}} - \frac{\sqrt{2}}{2R}\sqrt{1+\frac{\rho^2+\xi^2-1}{R^2}} \tag{3-A}$$

To calculate $J_2^1(\rho,\xi)$, first, the following integral is calculated.

$$\begin{aligned}
&\int_0^\infty e^{-\xi p} J_n(\rho p)dp = \frac{1}{\pi}\int_0^\pi \int_0^\infty e^{-\xi p}\cos(n\theta - \rho p \sin\theta)dp\,d\theta = \\
&\frac{1}{\pi}\int_0^\pi \int_0^\infty e^{-\xi p}\cos(n\theta)\cos(\rho p \sin\theta)dp\,d\theta + \frac{1}{\pi}\int_0^\pi \int_0^\infty e^{-\xi p}\sin(n\theta)\sin(\rho p\sin\theta)dp\,d\theta = \\
&\frac{1}{\pi}\int_0^\pi \frac{-\xi\cos(n\theta)}{\xi^2+\rho^2\sin^2\theta}d\theta + \frac{1}{\pi}\int_0^\pi \frac{\rho\sin\theta\sin(n\theta)}{\xi^2+\rho^2\sin^2\theta}d\theta
\end{aligned} \tag{4-A}$$

Considering the $(n=1)$

$$Z_2^1(\rho,\xi) = \int_0^\infty e^{-\xi p} J_1(\rho p)dp = \frac{1}{\rho}\left(\frac{\xi}{\sqrt{\xi^2+\rho^2}} - 1\right) \tag{5-A}$$

Therefore, by substituting $(\xi + i)$ in Eq. (5-A) and calculating the real part

$$J_2^1(\rho,\xi)=\frac{\sqrt{2}}{2\rho R}\left(\xi\sqrt{1+\frac{\rho^2+\xi^2-1}{R^2}}+\sqrt{1-\frac{\rho^2+\xi^2-1}{R^2}}\right)-\frac{\xi}{\rho\sqrt{\rho^2+\xi^2}} \quad \text{(6-A)}$$

$Z_1^1(\rho,\xi)$ is obtained by applying Eq. (5-A) and integrating ξ

$$Z_1^1(\rho,\xi)=\int_0^\infty \frac{e^{-\xi p}}{p}J_1(\rho p)dp=\frac{1}{\rho}\left(\sqrt{\xi^2+\rho^2}-\xi\right) \quad \text{(7-A)}$$

In the forgoing equation, by substituting $(\xi+i)$ for (ξ), $C_1^1(\rho,\xi)$ is calculated by considering the real part of the expression

$$J_1^1(\rho,\xi)=\xi+\sqrt{\xi^2+\rho^2}-\frac{\sqrt{2}}{2}R\sqrt{1+\frac{\rho^2+\xi^2-1}{R^2}} \quad \text{(8-A)}$$

By integrating both sides of the Eq. (1-A) of ξ, substituting $(\xi+i)$ for (ξ) and considering the real part of the equation, $C_1^0(\rho,\xi)$ is obtained.

$$J_1^0(\rho,\xi)=\frac{1}{2}\ln\left(\frac{R^2+R\sqrt{2}\left(\xi\sqrt{1+\frac{\rho^2+\xi^2-1}{R^2}}+\sqrt{1-\frac{\rho^2+\xi^2-1}{R^2}}\right)+\left(1+\xi^2\right)}{\left(\xi+\sqrt{\rho^2+\xi^2}\right)^2}\right) \quad \text{(9-A)}$$

Also, $J_0^1(\rho,\xi)$ is calculated by integrating both sides of Eq. (7-A) of ξ; then, by substituting $(\xi+i)$ for (ξ), the real part of equation is considered[23].
